# Pulmonary toxicity screening studies in male rats with TiO_2 _particulates substantially encapsulated with pyrogenically deposited, amorphous silica

**DOI:** 10.1186/1743-8977-3-3

**Published:** 2006-01-26

**Authors:** DB Warheit, TR Webb, KL Reed

**Affiliations:** 1 DuPont Haskell Laboratory for Health and Environmental Sciences, Newark, DE, USA

## Abstract

The aim of this study was to evaluate the acute lung toxicity in rats of intratracheally instilled TiO_2 _particles that have been substantially encapsulated with pyrogenically deposited, amorphous silica. Groups of rats were intratracheally instilled either with doses of 1 or 5 mg/kg of hydrophilic Pigment A TiO_2 _particles or doses of 1 or 5 mg/kg of the following control or particle-types: 1) R-100 TiO_2 _particles (hydrophilic in nature); 2) quartz particles, 3) carbonyl iron particles. Phosphate-buffered saline (PBS) instilled rats served as additional controls. Following exposures, the lungs of PBS and particle-exposed rats were evaluated for bronchoalveolar lavage (BAL) fluid inflammatory markers, cell proliferation, and by histopathology at post-instillation time points of 24 hrs, 1 week, 1 month and 3 months.

The bronchoalveolar lavage results demonstrated that lung exposures to quartz particles, at both concentrations but particularly at the higher dose, produced significant increases vs. controls in pulmonary inflammation and cytotoxicity indices. Exposures to Pigment A or R-100 TiO_2 _particles produced transient inflammatory and cell injury effects at 24 hours postexposure (pe), but these effects were not sustained when compared to quartz-related effects. Exposures to carbonyl iron particles or PBS resulted only in minor, short-term and reversible lung inflammation, likely related to the effects of the instillation procedure.

Histopathological analyses of lung tissues revealed that pulmonary exposures to Pigment A TiO_2 _particles produced minor inflammation at 24 hours postexposure and these effects were not significantly different from exposures to R-100 or carbonyl iron particles. Pigment A-exposed lung tissue sections appeared normal at 1 and 3 months postexposure. In contrast, pulmonary exposures to quartz particles in rats produced a dose-dependent lung inflammatory response characterized by neutrophils and foamy (lipid-containing) alveolar macrophage accumulation as well as evidence of early lung tissue thickening consistent with the development of pulmonary fibrosis.

Based on our results, we conclude the following: 1) Pulmonary instillation exposures to Pigment A TiO_2 _particles at 5 mg/kg produced a transient lung inflammatory response which was not different from the lung response to R-100 TiO_2 _particles or carbonyl iron particles; 2) the response to Pigment A was substantially less active in terms of inflammation, cytotoxicity, and fibrogenic effects than the positive control particle-type, quartz particles. Thus, based on the findings of this study, we would expect that inhaled Pigment A TiO_2 _particles would have a low risk potential for producing adverse pulmonary health effects.

## Introduction

This study was designed as a preliminary screen to determine whether Pigment A TiO_2 _particles (TiO_2 _particles that have been substantially encapsulated with pyrogenically deposited, amorphous silica) impart significant toxicity in the lungs of rats, and more importantly, how the activity of this TiO_2 _formulation compares with other reference particulate materials. Thus, the aim was to assess in rats, using a well-developed, short-term pulmonary bioassay the acute pulmonary toxicity effects of intratracheally instilled, Pigment A TiO_2 _particle samples and to compare the lung toxicity of these samples with 2 low toxicity particulate-types (negative controls) and a cytotoxic particulate (positive control) sample; and 2) to bridge the results of these instillation studies with data previously generated from inhalation studies with quartz particles in the form of crystalline silica and with carbonyl iron particles as the inhalation/instillation bridge materials.

## Methods

### Animals

Groups of male Crl:CD^®^(SD)IGSBR rats (Charles River Laboratories, Inc., Raleigh, North Carolina) were used in this study. The rats were approximately 8 weeks old at study start (mean weights in the range of 240 – 255 grams). All procedures using animals were reviewed and approved by the Institutional Animal Care and Use Committee and the animal program is fully accredited by the Association for Assessment and Accreditation of Laboratory Animal Care (AAALAC).

### Particle-types

Quartz particles (crystalline silica, Min-U-Sil 5) ranging in size from 1–3 μm were obtained from Pittsburgh Glass and Sand Corporation. Carbonyl iron (CI) particles ranging in size from 0.8 – 3.0 μm were obtained from GAF Corporation. R-100 titanium dioxide particles (~99 wt% titanium dioxide, ~1 wt% alumina) possessing an average particle size of ~300 nm and an average BET surface area of ~6 m^2^/g were obtained from the DuPont Company. Chloride process produced Pigment A titanium dioxide particles (~96 wt% titanium dioxide, ~1 wt% alumina, ~3 wt% amorphous silica [particle encapsulating]) possessing an average particle size of ~290 nm and an average BET surface area of ~7.9 m^2^/g were also obtained from the DuPont Company (see Table 1). Note that both of the DuPont-derived titanium dioxide samples were in the rutile form (crystal structure).

**Table 1:** Characterization of TiO_2 _and quartz particulates

d50			
		1° particle size	surface area

R-100	Rutile	300 nm	6 m^2^/g
Pigment A	Rutile	290 nm	~8 m^2^/g
Carbonyl Iron		~1.2 μm	N.D
Min-U-Sil Quartz	Crystalline	~1.5 μm	4 m^2^/g

N.D. = not determined

### General experimental design (see Additional file 1)

The fundamental features of this pulmonary bioassay are 1) dose response evaluation, and 2) time course assessments to determine the sustainability of any observed effect. Thus, the major endpoints of this study were the following: 1) time course and dose/response intensity of pulmonary inflammation and cytotoxicity; 2) airway and lung parenchymal cell proliferation; and 3) histopathological evaluation of lung tissue.

Groups of rats were intratracheally instilled with single doses of 1 or 5 mg/kg quartz (crystalline silica) particles, carbonyl iron particles, R-100 TiO_2 _particles, or Pigment A TiO_2 _particles. The intratracheal instillation route of entry technique is not a substitute for the more physiologically relevant inhalation method of exposure. It should be noted however, that pulmonary screening studies are not surrogates for more physiologically relevant, inhalation toxicity studies, such as 4-week inhalation, 90-day inhalation studies, or 2-year inhalation bioassay studies. However, the intratracheal instillation method of exposure can be a qualitatively reliable screen for assessing the pulmonary toxicity of particles [1,2]. All particles were prepared in a volume of phosphate-buffered saline (PBS) and subjected to polytron dispersement. Groups of PBS-instilled rats served as controls. The lungs of PBS, and particle-exposed rats were evaluated by bronchoalveolar lavage fluid analyses at 24 hr, 1 week, 1 month and 3 months postexposure (pe). For lung cell proliferation and histopathology studies, additional groups of animals were instilled with the particle-types listed above as well as PBS.

For the bronchoalveolar lavage studies, 5 male rats/group were exposed via intratracheal instillation to 1) vehicle control – Phosphate-buffered saline (PBS); 2) carbonyl iron particles in PBS at 1 or 5 mg/kg; 3) hydrophilic R-100 TiO_2 _particles in PBS at 1 or 5 mg/kg; 4) hydrophilic Pigment A TiO_2 _particles in PBS at 1 or 5 mg/kg; or 5) Min-U-Sil crystalline quartz particles in PBS at 1 or 5 mg/kg (see Table 1 and Additional file 1).

For the lung tissue studies, additional groups of animals (4 rats/group) were instilled with the particle-types listed above plus the vehicle control, i.e., PBS. These studies and corresponding groups of rats were dedicated to lung tissue analyses but only the high dose groups (5 mg/kg) and PBS controls were utilized in the morphology studies. These studies consisted of cell proliferation assessments and histopathological evaluations of the lower respiratory tract. Similar to the BAL fluid studies, the intratracheal instillation exposure period was followed by 24-hour, 1-week, 1-month, and 3-month recovery periods.

### Pulmonary lavage

The lungs of sham and particulate-exposed rats were lavaged with a warmed phosphate-buffered saline (PBS) solution as described previously. Methodologies for cell counts, differentials and pulmonary biomarkers in lavaged fluids were conducted as previously described [3,4]. Briefly, the first 12 mL of lavaged fluids recovered from the lungs of PBS or particulate-exposed rats was centrifuged at 700 g, and 2 mL of the supernatant was removed for biochemical studies. All biochemical assays were performed on BAL fluids using a Roche Diagnostics (BMC)/Hitachi^® ^717 clinical chemistry analyzer using Roche Diagnostics (BMC)/Hitachi^® ^reagents. Lactate dehydrogenase (LDH), alkaline phosphatase (ALP), and lavage fluid protein were measured using Roche Diagnostics (BMC)/Hitachi^® ^reagents. Lactate dehydrogenase is a cytoplasmic enzyme and is used as an indicator of cell injury. Alkaline phosphatase activity is a measure of Type II alveolar epithelial cell secretory activity, and increased ALP activity in BAL fluids is considered to be an indicator of Type II lung epithelial cell toxicity. Increases in BAL fluid micro protein (MTP) concentrations generally are consistent with enhanced permeability of vascular proteins into the alveolar regions, indicating a breakdown in the integrity of the alveolar-capillary barrier.

### Pulmonary cell proliferation studies

Groups of particulate-exposed rats and corresponding controls were pulsed 24 hrs after instillation, as well as 1 week, 1 and 3 months postexposure, with an intraperitoneal injection of 5-bromo-2'deoxyuridine (BrdU) dissolved in a 0.5 N sodium bicarbonate buffer solution at a dose of 100 mg/kg body weight. The animals were euthanized 6 hrs later by pentobarbital injection. Following cessation of spontaneous respiration, the lungs were infused with a neutral buffered formalin fixative at a pressure of 21 cm H_2_O. After 20 minutes of fixation, the trachea was clamped, and the heart and lungs were carefully removed *en bloc *and immersion-fixed in formalin. In addition, a 1-cm piece of duodenum (which served as a positive control) was removed and stored in formaldehyde. Subsequently, parasagittal sections from the right cranial and caudal lobes and regions of the left lung lobes as well as the duodenal sections were dehydrated in 70% ethanol and sectioned for histology. The sections were embedded in paraffin, cut, and mounted on glass slides. The slides were stained with an anti-BrdU antibody, with an AEC (3-amino-9-ethyl carbazole) marker, and counter-stained with aqueous hematoxylin. A minimum of 1000 cells/animal were counted each in terminal bronchiolar and alveolar regions. For each treatment group, immunostained nuclei in airways (i.e., terminal bronchiolar epithelial cells) or lung parenchyma (i.e., epithelia, interstitial cells or macrophages) were counted by light microscopy at × 1000 magnification [3,4].

### Lung histopathology studies

The lungs of rats exposed to particulates or PBS controls were prepared for microscopy by airway infusion under pressure (21 cm H_2_O) at 24 hours, 1 week, 1 and 3 months postexposure. Sagittal sections of the left and right lungs were made with a razor blade. Tissue blocks were dissected from left, right upper, and right lower regions of the lung and were subsequently prepared for light microscopy (paraffin embedded, sectioned, and hematoxylin-eosin stained) [3,4].

### Statistical analyses

For analyses, each of the experimental values were compared to their corresponding sham control values for each time point. A one-way analysis of variance (ANOVA) and Bartlett's test were calculated for each sampling time. When the F test from ANOVA was significant, the Dunnett test was used to compare means from the control group and each of the groups exposed to particulates. Significance was judged at the 0.05 probability level.

## Results

### Lung weights

Lung weights of rats were enhanced with increasing age on the study (i.e., increased postexposure time periods following instillation). Lung weights in high dose quartz-exposed rats were slightly increased vs. controls at 1 week, and at 1 month and 3 months postexposure (data not shown).

### Bronchoalveolar lavage fluid results

#### Pulmonary inflammation

**Figure 1:**
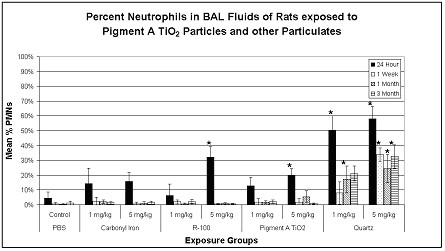
Pulmonary inflammation in particulate-exposed rats and controls as evidenced by % neutrophils (PMN) in BAL fluids at 24 hrs, 1 week, 1 month and 3 months postexposure (pe). Values given are means ± S.D. Intratracheal instillation exposures of several particle-types produced a short-term, pulmonary inflammatory response, as evidenced by an increase in the percentages/numbers of BAL-recovered neutrophils, measured at 24 hrs postexposure. However, only the exposures to quartz particles (1 and 5 mg/kg) produced sustained pulmonary inflammatory responses, as measured through 3 months postexposure. *p < 0.05.

The numbers of cells recovered by bronchoalveolar lavage from the lungs of high dose quartz-exposed (5 mg/kg) groups were substantially higher than any of the other groups for all postexposure time periods (data not shown). Intratracheal instillation exposures of several particle-types produced a short-term, pulmonary inflammatory response, as evidenced by an increase in the percentages/numbers of BAL-recovered neutrophils, measured at 24 hrs postexposure. However, only the exposures to quartz particles (1 and 5 mg/kg) produced sustained pulmonary inflammatory responses, as measured through 3 months postexposure (Fig. 1).

#### BAL fluid parameters

**Figure 2:**
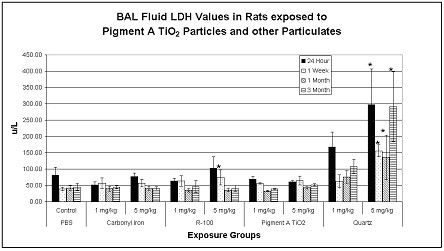
BAL fluid LDH values for particulate-exposed rats and corresponding controls at 24 hrs, 1 week, 1 month and 3 months postexposure (pe). Values given are means ± S.D. Transient and reversible increases in BAL fluid lactate dehydrogenase values were measured in the lungs of high dose (5 mg/kg) R-100 TiO_2_-exposed rats at 1 week postexposure. In contrast, exposures to 5 mg/kg quartz particles produced a sustained increase in BAL fluid LDH values through the 3-month postexposure period. *p < 0.05.

**Figure 3:**
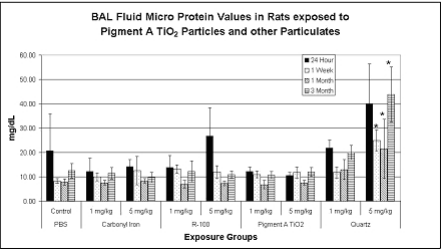
BAL fluid protein (MTP) values for particulate-exposed rats and corresponding controls at 24 hrs, 1 week, 1 month and 3 months postexposure. Values given are means ± S.D. Transient increases in BAL fluid microprotein values were measured in the lungs of high dose (5 mg/kg) TiO_2_-exposed rats at 24 hrs postexposure, but were not different from controls at 1 week postexposure. In contrast, exposures to 5 mg/kg quartz particles produced a sustained increase in BAL fluid microprotein values at 24 hrs, 1 week, 1 and 3 months postexposure. * p < 0.05.

Transient and reversible increases in BAL fluid lactate dehydrogenase values, as an indicator of cytotoxicity, were measured in the lungs of high dose (5 mg/kg) R-100 exposed rats at 1 week postexposure, but were not sustained through the other postexposure time periods. In contrast, exposures to 5 mg/kg quartz particles produced a sustained increase in BAL fluid LDH values through the 3-month postexposure period (Fig. 2). Transient increases in BAL fluid microprotein (MTP) values were measured in the lungs of high dose (5 mg/kg) R-100-exposed rats at 24 hrs postexposure, but were not different from controls at 1 week postexposure. In contrast, exposures to 5 mg/kg quartz particles produced a sustained increase in BAL fluid microprotein values at 24 hrs, 1 week, 1 and 3 months postexposure (Fig. 3). Transient increases in BAL fluid alkaline phosphatase values were measured only in the lungs of R-100-exposed rats at 1 week postexposure (5 mg/kg), but significant increases in BAL fluid alkaline phosphatase values were measured at 1 week through 3 months postexposure in rats exposed to 5 mg/kg quartz particles (data not shown).

To summarize the results from BAL fluid biomarker studies, pulmonary exposures to quartz particles produced a sustained, dose-dependent, lung inflammatory response, concomitant with cytotoxic effects, measured from 24 hrs through 3 months postexposure. Exposures to R-100 particles (5 mg/kg) produced a small but transient pulmonary inflammatory response, but this effect was not sustained. Exposures to carbonyl iron particles or to Pigment A TiO_2 _particles produced a brief neutrophilic response at 24 hrs postexposure, however, this was likely related to the instillation exposure methodology.

### Lung cell proliferation and histopathology studies

#### Cell proliferation results

Tracheobronchial cell proliferation rates (% immunostained cells taking up BrdU) were measured in high dose (5 mg/kg), particulate-exposed rats and corresponding controls at 24 hrs, 1 week, and 1 and 3 months postexposure (pe). Although increases in cell labeling indices were noted in R-100 and quartz-exposed animals at 24 hrs postexposure, these effects were not sustained (data not shown).

**Figure 4:**
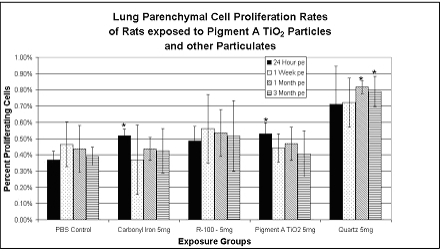
Lung parenchymal cell proliferation rates (BrdU) in particulate-exposed rats and corresponding controls at 24 hrs, 1 week, 1 month and 3 months postexposure (pe). Values given are means ± S.D. Small but significant transient increases in lung cell proliferation indices were measured in the carbonyl iron, Pigment A TiO_2_-exposed, and quartz-exposed rats at 24 hrs but were not sustained. Significantly larger increases in cell proliferation indices were measured in the lungs of quartz exposed rats through 3 months postexposure. *p < 0.05

Lung parenchymal cell proliferation rates (% immunostained cells taking up BrdU) were measured in high dose (5 mg/kg), particulate-exposed rats and corresponding controls at 24 hrs, 1 week, and 1 and 3 months postexposure (pe). Small but significant transient increases in lung cell proliferation indices were measured in the carbonyl iron or Pigment A TiO_2_-exposed rats at 24 hrs but were not sustained. Significantly larger increases in cell proliferation indices were measured in the lungs of quartz exposed rats through 3 months postexposure. (Fig. 4).

In summary, exposures to 5 mg/kg quartz particles produced increased tracheobronchial cell proliferation compared to PBS controls, but increases were statistically significant only at 24 hrs postexposure. However, exposures to 5 mg/kg quartz particles produced substantially greater lung parenchymal cell proliferation rates at all time points postexposure, suggesting a greater likelihood to result in lung cell mutations over time with continued exposures.

### Histopathological evaluation

**Figure 5:**
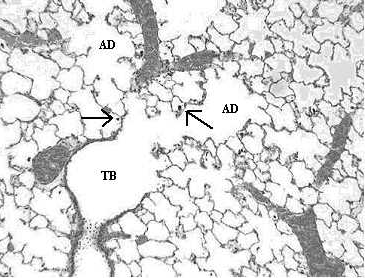
Light micrograph of lung tissue from a rat exposed to R-100 TiO_2 _particles (5 mgs/kg) at 1 day post-instillation exposure. This micrograph illustrates the terminal bronchiole (TB) and corresponding alveolar ducts (AD), and demonstrates normal lung architecture and normal macrophage phagocytosis of R-100 particles (arrows). Magnification = ×100.

**Figure 6:**
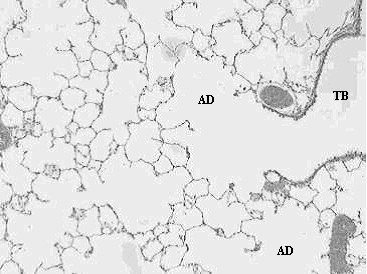
Light micrograph of lung tissue of a rat exposed to Pigment A TiO_2 _particles (5 mg/kg) at 3 months post-instillation exposure. This micrograph illustrates the terminal bronchiole (TB) and corresponding alveolar ducts (AD), and demonstrates normal lung architecture, indicating that exposure to Pigment A TiO_2 _particles produced no adverse pulmonary effects. Magnification = ×100.

Histopathological analyses of lung tissues revealed that pulmonary exposures to carbonyl iron, to R-100 particles, or to Pigment A TiO_2 _particles in rats produced no significant adverse effects when compared to PBS-exposed controls, as evidenced by the normal lung architecture observed in the exposed animals at post-instillation exposure time periods ranging from 24 hours to 3 months (Figs. 5, 6). Histopathological analyses of lung tissues revealed no differences between the R-100 TiO_2_-exposed rats vs. those exposed to Pigment A TiO_2 _particles (Figs. 5, 6). A light micrograph of a lung tissue section of a rat instilled with 5 mg/kg R-100 TiO_2 _particles at 24 hrs postexposure demonstrated deposition of instilled particles and normal alveolar macrophage phagocytic responses (Fig. 5). Lung tissue sections from rats instilled with 5 mg/kg R-100 TiO_2 _particles appeared very similar histologically to the lung tissue sections from the Pigment A TiO_2_-exposed rats at each postexposure time period and demonstrated normal pulmonary architecture.

**Figure 7:**
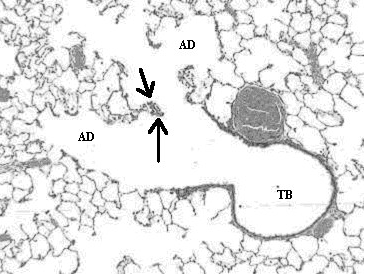
Light micrograph of lung tissue from a rat exposed to quartz particles (5 mg/kg) at 1 month post-instillation exposure. This micrograph illustrates the terminal bronchiole (TB) and corresponding alveolar ducts (AD). Note the prominence of tissue thickening (arrows) at the junction at the terminal bronchiole and alveolar duct bifurcation. Magnification = ×100.

**Figure 8:**
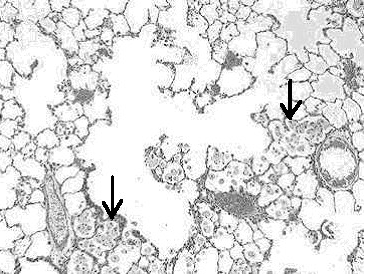
Light micrograph of lung tissue from a rat exposed to quartz particles (5 mg/kg) at 3 months post-instillation exposure. Note the tissue accumulation of foamy multinucleated alveolar macrophages (arrows) within alveolar spaces. The macrophages have migrated to the sites of quartz particle deposition at the terminal bronchiolar alveolar junctions. The accumulation of lipid-filled macrophages and lack of clearance is a common feature of the progressive nature of silica induced lung disease. Magnification = ×100.

**Figure 9:**
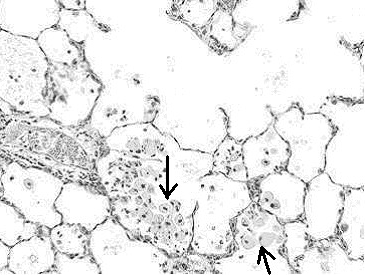
Higher magnification light micrograph of lung tissue from a rat exposed to quartz particles (5 mg/kg) at 3 months post-instillation exposure. Note the tissue accumulation of foamy multinucleated alveolar macrophages (arrows) within alveolar spaces. This is a common feature of the progressive nature of silica induced lung disease. Magnification = ×200.

Histopathological analyses of lung tissues revealed that pulmonary exposures to quartz particles in rats produced dose-dependent lung inflammatory responses characterized by neutrophils and foamy (lipid-containing) alveolar macrophage accumulation. In addition, lung tissue thickening as a prelude to the development of fibrosis was evident and progressive (Figs. 7, 8, 9).

## Discussion

The objective of this study was to assess the acute lung toxicity of intratracheally instilled, hydrophilic Pigment A TiO_2 _particles in rats. Using pulmonary bioassay methodology, the pulmonary toxicity of instilled Pigment A TiO_2 _particles was compared with a positive control particle-type, quartz, as well as two negative control particle-types, carbonyl iron particles and R-100 TiO_2 _particles.

Pigment A TiO_2, _R-100 TiO_2_, or CI did not produce significant adverse pulmonary effects in any of the bioassays in this study (BAL inflammatory indicators, cell proliferation, or histopathology). These particle-types produced only a transient pulmonary inflammatory effect. Because this inflammatory effect was also observed in the PBS vehicle control group, one may assume that the effect is a result of the instillation process and not of the particles in the lung per se.

In contrast, quartz particles, particularly at the higher dose, produced significant adverse effects in pulmonary inflammation, cytotoxicity and lung parenchymal cell proliferation endpoints, all of which continued through the 3-month postexposure study period. Histopathological evaluation further demonstrated that quartz particles produced pulmonary inflammation, foamy macrophage and tissue thickening (as a prelude to fibrosis).

Based upon the data generated from this pulmonary bioassay, we conclude that hydrophilic Pigment A TiO_2 _particles do not cause significant pulmonary toxicity and the pulmonary effects measured are not significantly different from the effects produced by hydrophilic R-100 TiO_2 _particles.

In an earlier study, we assessed the acute pulmonary toxicity potential in rats of a series of intratracheally instilled TiO_2 _particle-types (2 or 10 mg/kg) possessing (1) no surface treatment, (2) an alumina-only surface treatment (~3.2 wt% Al_2_O_3 _equivalent) or (3) a combined alumina/silica surface treatment (~1.8–5.8 wt% Al_2_O_3 _equivalent, ~3.0–10.5 wt% SiO_2 _equivalent). Included in this study were hydrophilic TiO_2 _particles, codified as R-100, which possessed no inorganic surface treatment but did possess a small quantity of an organic surface treatment, namely, triethanolamine. Note that the inhalation toxicity potential of the pigment possessing limited treatment (i.e., 1% Al_2_O_3_) had previously been evaluated, a fact that allowed us to bridge the results of this evaluation with those derived from the above mentioned instillation toxicity study. During that study, saline-instilled rats served as controls. The lungs of sham and exposed rats were evaluated by bronchoalveolar lavage at 24 hr, 1 week, 1 and 3 months postexposure (pe). The results demonstrated that the high dose (10 mg/kg) pigment grade TiO_2 _particles possessing the greatest amount of inorganic surface treatment (Al_2_O_3_/SiO_2 _combination) produced a more intense lung inflammatory response relative to the other samples. This effect was measured only through 1 month but not the 3-month postexposure period, indicating that the effect of the heavily surface treated TiO_2 _particle (i.e., 84% TiO_2_, ~6% alumina and ~10% amorphous silica) was relatively minor when compared to the effects of quartz particles [2].

These results are similar to the findings in another previous study evaluating surface treatments, wherein we assessed the toxicity of pigment-grade titanium dioxide particles made hydrophobic by surface application of octyltriethoxysilane (OTES) [5]. In similar-type pulmonary bioassay studies, at higher doses (2 and 10 mg/kg), the toxicity of OTES-coated TiO_2 _particles was not significantly different from the hydrophilic R-100 TiO_2 _particles. The R-100 has a mean particle size of 300 nm and a surface area of ~6 m^2^/g, while the OTES-treated TiO_2 _particle has a primary particle size of 230 nm and a surface area of ~8 m^2^/g.

Surface treatments on TiO_2 _particles have been a subject of interest over the past few years. The potential concern for the effects of hydrophobic coatings was initially raised by Pott and coworkers [6,7]. These investigators reported that exposures to hydrophobic-coated, ultrafine (~20–30 nm) titanium dioxide particles (T-805 sample) produced unexpected lethality in intratracheally exposed rats [6,7]. Pott reported that the first two rats which were treated with 6 mg hydrophobic ultrafine TiO_2 _particles (T-805) demonstrated immediate symptoms of respiratory distress when compared to the rats similarly exposed to other particulates; and they survived less than one-half hour; instillation of 3 mg T-805 particles also induced a fatal effect, and 1 mg doses were tolerated with some limitations. Subsequent studies were conducted with weekly intratracheal instillations of 0.5 mg doses of hydrophobic TiO_2 _(T-805), and still produced some mortality. In contrast, Pott reported that nearly all of the rats exposed to equivalent dosages of hydrophilic, ultrafine titanium dioxide particles (P-25 sample) (similar particle sizes) survived the intratracheal instillation exposures. A confounding factor of the Pott study which had not been addressed or properly controlled for was the potential toxicity of 1% Tween, which was added as a detergent selectively to the T-805 sample but not to the hydrophilic P-25 sample, creating an additional variable in the study. Thus, it was conceivable that the detergent significantly contributed to the toxic effects observed in the T-805-exposed rats.

It is important to note that the findings reported by Pott and colleagues have not been confirmed by a variety of other investigators who have compared the pulmonary hazard effects of hydrophilic and hydrophobic coated titanium dioxide particles. As discussed above, we have previously evaluated in rats the pulmonary toxicity of instilled hydrophilic vs. hydrophobic, pigment-grade TiO_2 _particles, using a pulmonary bioassay methodology. The results demonstrated that only the high-dose (10 mg/kg) hydrophilic, pigment-grade TiO_2 _particles and those with particle-types containing a surfactant, viz. Tween 80, produced a transient and reversible pulmonary inflammatory response, and this was resolved within 1 week postexposure. In that study, we concluded that the OTES hydrophobic coating on the pigment-grade TiO_2 _particle does not cause significant pulmonary toxicity.

Studies reported by other investigators have confirmed these results. In this regard, Hohr et al. [8] assessed the acute pulmonary inflammation in rats after intratracheal instillation of surface modified (hydrophilic and hydrophobic) fine (180 nm) and ultrafine (20–30 nm) TiO_2 _particles at equivalent mass (1 or 6 mg) or calculated surface area doses (100, 500, 600, and 3000 cm^2^). These investigators concluded that BAL fluid biomarkers of lung inflammatory responses correlated with the administered surface dose delivered to the lungs. Moreover, the hydrophobic-coated TiO_2 _particles produced decreased lung inflammation relative to hydrophilic-coated particulates, however, these minor effects were not significantly different between the two samples. The conclusions drawn from this study was that the surface area rather than the hydrophobic surface coating influences the acute pulmonary inflammatory response produced following intratracheal instillation of either fine grade or ultrafine-sized TiO_2 _particles.

In another study comparing effects of surface coatings, Oberdorster [9] exposed rats via intratracheal instillation to two different types of aggregated ultrafine TiO_2 _particle samples (particle size of both types reported to be ~20 nm) at doses of 50 or 500 μg. One type was silane-coated, making the particle surface hydrophobic, while the other particle sample was uncoated and hydrophilic. Hydrophobic-coated ultrafine TiO_2 _particles produced a reduced pulmonary inflammatory response at 24 hours postexposure when compared to identical doses of the uncoated, hydrophilic TiO_2 _particles. Oberdorster concluded that his findings appear to conflict with an earlier report by Pott and coworkers [6,7], who, as discussed above, reported that larger doses of instilled hydrophobic- coated, but not uncoated ultrafine TiO_2 _particles were acutely toxic and produced mortality in rats.

Rehn and colleagues [10] investigated lung inflammation and genotoxic effects of two types of ultrafine titanium dioxide particles. The lungs of female rats were exposed by intratracheal instillation to a range of doses of uncoated (P-25) or hydrophobic-coated (T-805) ultrafine TiO_2 _particles and assessed at 3, 21 and 90 days following instillation exposures. Quartz particles (DQ12) and saline were utilized as positive and negative controls, respectively. Pulmonary inflammatory responses of sham and particle-exposed rats were assessed using bronchoalveolar lavage biomarkers and the genotoxic analyses were conducted by immunohistochemical assessments of 8-oxoguanine in lung tissue. Quartz exposures produced persistent lung inflammatory responses, measured through 90 days postexposure. In contrast, no pulmonary inflammation was evident in rats exposed to either form of TiO_2 _particle-types when measured 90 days postexposure. The investigators concluded that the both the uncoated and hydrophobic coated TiO_2 _produced no significant adverse pulmonary effects.

The results of numerous studies demonstrate that inhalation exposures to pigment-grade TiO_2 _particles or carbonyl iron particles in rats produces low pulmonary toxicity, and induces adverse inflammatory effects only at substantial particle overload concentrations [4,11-15]. In the current study, the highest dose of instilled hydrophilic, Pigment A TiO_2 _particles (5 mg/kg) or carbonyl iron particles produced only a minor, transient lung inflammatory response, measured at 24 hrs postexposure, and this was related primarily to the route of exposure. Thus, the inhalation toxicity data and the instillation toxicity results for hydrophilic, R-100 TiO_2 _particles and carbonyl iron particles in rats are consistent – i.e., clearly demonstrating that pulmonary exposures produce few adverse lung effects. These lung toxicity results assessed following TiO_2 _particulate exposures in rats clearly contrast with the pulmonary effects measured following inhalation [3] or intratracheal instillation exposures in rats [16] to crystalline silica particles, which produce persistent pulmonary inflammatory responses leading to the development of lung fibrosis, in both inhalation and instillation models.

In summary, using a pulmonary bioassay and bridging methodology, the acute lung toxicity of intratracheally instilled hydrophilic, Pigment A TiO_2 _particulates was compared with a positive control particle-type, quartz, as well as with 2 negative control particle-types, namely carbonyl iron particles and hydrophilic, R-100 TiO_2 _particles. In addition, the results of these instillation studies were bridged with data previously generated from inhalation studies with quartz, carbonyl iron particles and pigment-grade R-100 hydrophilic titanium dioxide particles. The results presented herein demonstrate that intratracheally instilled, 5 mg/kg doses of Pigment A TiO_2 _particles do not produce significant lung toxicity in rats; results similar to those derived from R-100 titanium dioxide samples. At similar doses, exposures to quartz particles produces a sustained pulmonary inflammatory response in rats, leading to the development of pulmonary fibrosis and other adverse lung effects. Accordingly, based on the findings of this study, it is expected that inhaled Pigment A TiO_2 _particles would have a low risk potential for producing adverse pulmonary health effects.

## Supplementary Material

Additional File 1 Protocol for pigment A TiO_2 _particle bioassay study. Click here for file
