# Supplementary figures and images for: Pulmonary toxicity screening studies in male rats with TiO2 particulates substantially encapsulated with pyrogenically deposited, amorphous silica
Source: Part Fibre Toxicol. 2006 Jan 26;3:3. doi: 10.1186/1743-8977-3-3 (PMC1402317; doi:10.1186/1743-8977-3-3)

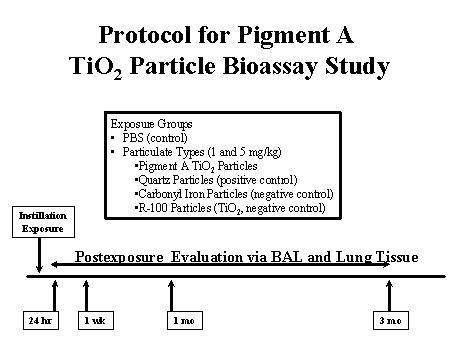

Supplement: Additional File 1 — Protocol for pigment A TiO2 particle bioassay study. [file 1743-8977-3-3-S1.jpeg]
